# Hospital Progress Abroad

**Published:** 1911-09-30

**Authors:** 


### Hospital Progress Abroad.

### GERMAN METHODS.

A correspondent wrote to the *Zeitschrift fur Krankenanstalten* to ask "whether or not there exists in the German Empire an institution for the training of hospital secretaries?" and the reply to this inquiry may be of interest to those who wish to study German methods. "There does not exist any such institution in Germany. Our hospital directors are in general officials appointed by the State or by individual Corporations, and they have to acquire official training. There is a school for officials (Beambtenschule) at Aschersleben. Special instruction in secretarial duties is given at the best hospitals, to which the newly-appointed superintendents are sent for a probationary period: for instance, in the Rhine Provinces to Superintendent Beyer at the Provincial Hospital, Bonn; in Prussia to Superintendent Diesener at Berlin (Urban Hospital); in Bavaria to Herr Schmidtler at the City Hospital, Munich, and other institutions whose managers are men of wide experience. There is a special institute for matrons at the *Anschar-Schwesternhause* at Kiel.

### AUSTRALIAN NOTES.

The foundation-stone of a new infirmary wing and septic ward at the Women's Hospital (Melbourne) was laid by Mrs. Burston, ex-Lady Mayoress, on February 1 last. The hospital was founded in 1856, and has sheltered over a quarter-million of patients since that date in very inadequate buildings for many years. Now public opinion is being educated, and the necessary funds are already nearly all in hand. The new wing is to be three storeys in height, the top floor being the septic ward with a flat roof as airing ground for convalescents.

According to the *Australasian Medical Gazette*, the Chief Secretary of New South Wales, in reply to a deputation from the Political Labour Conference, has expressed his personal opinion as being in favour of the nationalisation of hospitals when the public finances will permit of this being done. With this opinion the *Gazette* is not in agreement, and apart from the abuse of the hospitals by those well able to afford payment elsewhere, an abuse which, if rectified, would remove to a great extent the present congestion and frequent lack of funds, it can see at present no valid reason for "reforms" of such a sweeping nature. It hopes, however, that, should the question of nationalisation seriously came forward, the whole problem will be discussed solely on its merits, publicly and without party feeling.

